# Bio-inspired micro-to-nanoporous polymers with tunable stiffness

**DOI:** 10.3762/bjnano.8.92

**Published:** 2017-04-21

**Authors:** Julia Syurik, Ruth Schwaiger, Prerna Sudera, Stephan Weyand, Siegbert Johnsen, Gabriele Wiegand, Hendrik Hölscher

**Affiliations:** 1 Institute for Microstructure Technology, Karlsruhe Institute of Technology (KIT), Hermann-von-Helmholtz-Platz 1, 76344 Eggenstein-Leopoldshafen, Germany; 2 Institute for Applied Materials, Karlsruhe Institute of Technology (KIT), Hermann-von-Helmholtz-Platz 1, 76344 Eggenstein-Leopoldshafen, Germany; 3 Institute of Catalysis Research and Technology, Karlsruhe Institute of Technology (KIT), Hermann-von-Helmholtz-Platz 1, 76344 Eggenstein-Leopoldshafen, Germany

**Keywords:** biomimetics, polymeric materials, supercritical carbon dioxide (SC-CO_2_), tunable storage modulus

## Abstract

**Background:** Inspired by structural hierarchies and the related excellent mechanical properties of biological materials, we created a smoothly graded micro- to nanoporous structure from a thermoplastic polymer.

**Results:** The viscoelastic properties for the different pore sizes were investigated in the glassy regime by dynamic flat-punch indentation. Interestingly, the storage modulus was observed to increase with increasing pore-area fraction.

**Conclusion:** This outcome appears counterintuitive at first sight, but can be rationalized by an increase of the pore wall thickness as determined by our quantitative analysis of the pore structure. Therefore, our approach represents a non-chemical way to tune the elastic properties and their local variation for a broad range of polymers by adjusting the pore size gradient.

## Introduction

Functional adaptation through porous structures is widely present in nature [1]. Natural structures are often made of hierarchical porous networks, serving mainly for passive mechanical functions [1–3], but also for capillary channels and membranes [1]. Nature offers several examples of porous structures and composites having excellent mechanical properties that surpass the properties of the constituent materials. Prominent examples are wood, bone, or bird beaks [1]. The outstanding properties of the pomelo peel, though, are less known. The pomelo (*Citrus maxima*) is a citrus fruit, which survives falls from heights above 10 m without any visible external damage [2]. Its tens of millimetres thick protective peel with a hierarchical, graded porous structure was shown to be critical for this amazing mechanical performance [2,4]. Graded porous structures show great potential for impact-resistant components [2] and might also be beneficial to other functional surfaces that, for example, require a stiffness gradient. The recent review by Sahay et al. [5] on synthetic dry adhesives postulates that achieving stiffness gradients is one of the most important goals in improvement of re-usability and durability of the products.

Porous structures with a pore-size gradient have been manufactured in the past from different materials such as ceramics [6–8], metals [9], composites [10] and polymers [11]. These structures exhibited improved thermal [12] and mechanical properties [9]. Different techniques such as combining printing and infiltration [12], combined foaming and pyrolysis [8], or compression moulding of expandable beads [11] have been utilised. Recently, gradient metal-foam structures emulating the pomelo structure on the cellular level were manufactured using a modified, biomimetic investment-casting manufacturing technique [13]. The protective peel was transformed into amorphous silica by bio-templating down to the nanometre-scale, creating a biomorphous inorganic and, therefore, temperature-resistant gradient-foam material .

It is well known that polymeric foams have a lower density than the respective monolithic bulk material and, thus, typically exhibit a reduced elastic modulus [14]. Microporous polymers have been under development since the 1960s [15–16]. The development of fabrication methods allowing for smaller pore sizes made the next generation of nanoporous polymeric foams accessible [17]. Saturation in supercritical carbon dioxide (SC-CO_2_) [18] is a well-established method for the fabrication of polymeric foams. The process of pore nucleation by saturation in SC-CO_2_ consists of three basic steps [14,19]: (i) mixing of a polymer and SC-CO_2_ to form a homogeneous solution; (ii) pore nucleation and phase separation induced by a thermodynamic instability, which is usually caused by a temperature increase or a pressure decrease, and (iii) pore growth due to diffusion of SC-CO_2_ from the polymer matrix to the pores [20]. When the polymer is subjected to inhomogeneous pressure and/or temperature conditions during steps (ii) and (iii), the pore size within the polymer can vary [20].

To the best of our knowlegde, the correlation between the density of the porous material and its storage modulus, especially the size-dependent mechanical properties of a material with a pore-size gradient, have not been demonstrated yet. For nanocellular polymers, for example, two opposite effects were predicted: (i) local hardening of the material due to material confinement on nanometre dimensions [21] and (ii) a great reduction of elastic modulus of thin polymeric walls as predicted from the point of discontinuous molecular dynamics [22]. Therefore, in addition to the wide range of applications, the size-dependent viscoelastic properties of polymers with a morphological gradient, especially in the nanometre-range, are a very interesting phenomena.

Here, we demonstrate that a foam of a stiff polymer such as poly(methyl methacrylate) (PMMA) can exhibit a gradually changing effective elastic modulus when the local morphology of the sample undergoes a transition from microcellular to nanocellular. Porous PMMA films with a controlled gradient of the pore size were fabricated and investigated by dynamic flat-punch nanoindentation in order to obtain insight into the influence of the graded pore structure on the local viscoelastic properties.

## Experimental

### Materials and foaming process

**Figure 1:**
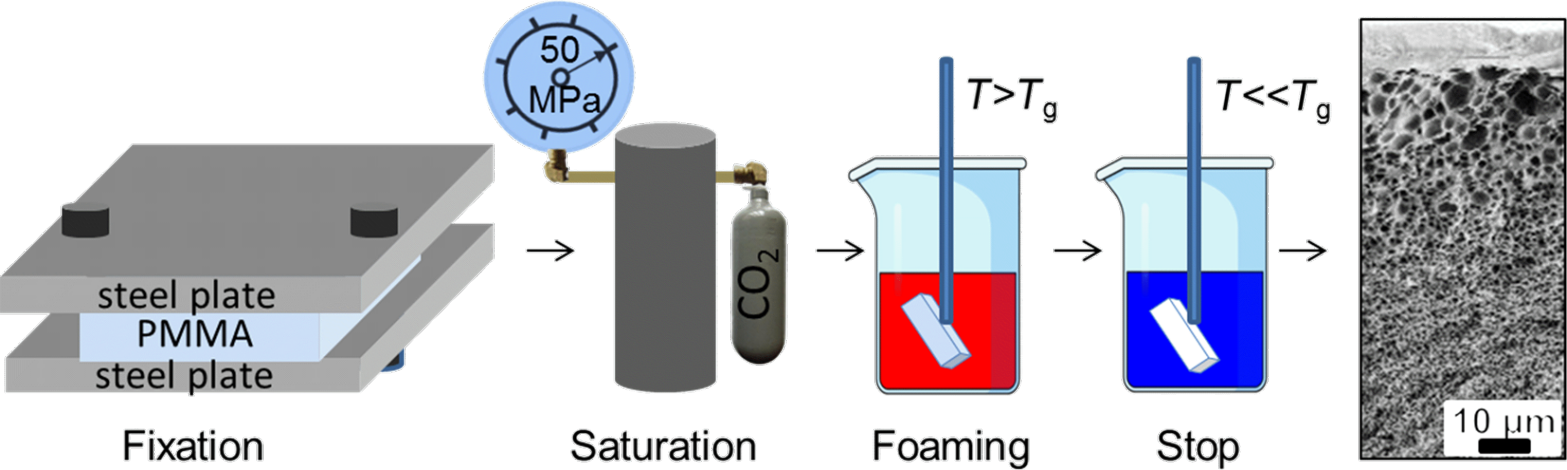
A schematic of the foaming process showing the critical steps to obtain a sample with a controlled gradient of pore size. The pore-size gradient is achieved through an inhomogeneous CO_2_ concentration in the sample at the saturation step and a temperature gradient during the pore growth. An SEM image of a cross section of a generic sample is shown. The average pore sizes range from 5 μm to 200 nm over the cross section.

Porous poly(methyl methacrylate) (PMMA) films were produced from PMMA (Topacryl AG, Switzerland) sheets of 500 μm thickness, with a glass-transition temperature (*T*_g_) of 105 °C (measured by thermogravimetric analysis (TGA) for the PMMA sheets) and a bulk elastic modulus of 3300 MPa. The as-purchased PMMA films were cut to 20 mm × 20 mm chips and clamped between two flat metal plates with four screws, so that the direction of diffusion was almost parallel to the sample plane (Figure 1). A process of pressure-induced saturation [20] with supercritical CO_2_ (Air Liquid, 99.99% purity) was employed. In order to reach saturation, the high-pressure cell was connected to a CO_2_ balloon and a pump, which can compress CO_2_ up to a pressure of 50 MPa (see Figure 1). For the process, a saturation pressure of 30 MPa and a temperature of 44 °C were applied for 4 h, followed by a rapid pressure quench. Directly after rapid depressurisation, the samples were heated to *T*_g_ in Antifrogen^®^ N (Clariant International Ltd., Switzerland), which contains mostly monopropylene glycol and is commonly used as a heat-transfer medium. A temperature gradient was employed by dipping the PMMA films, while they were clamped between the metal plates, into the hot liquid (105 °C) for 15 s. To stop the pore growth, the sample was cooled in deionised (DI) water with a temperature of 5 °C. No other chemical treatment was applied. Due to the expansion, the thickness of the sample after the foaming doubled to approximately 1 mm.

### Size-exclusion chromatography

The number-average molar mass (*M*_n_), mass-average molar mass (*M*_w_) and peak-maximum (*M*_p_) average molar mass of untreated and foamed samples were determined by size-exclusion chromatography (SEC, also known as gel-permeation chromatography, GPC) using an Agilent 1200 Series GPC-SEC system equipped with columns from Polymer Standards Service (PSS SDV Lux 5μm, 1000 Å and 10^5^ Å). We conducted the SEC measurements twice for each type of sample (pure, 4 μm, 200 nm) and observed the same overall results with negligible deviation between the two experiments.

### Analysis of morphology

**Figure 2:**
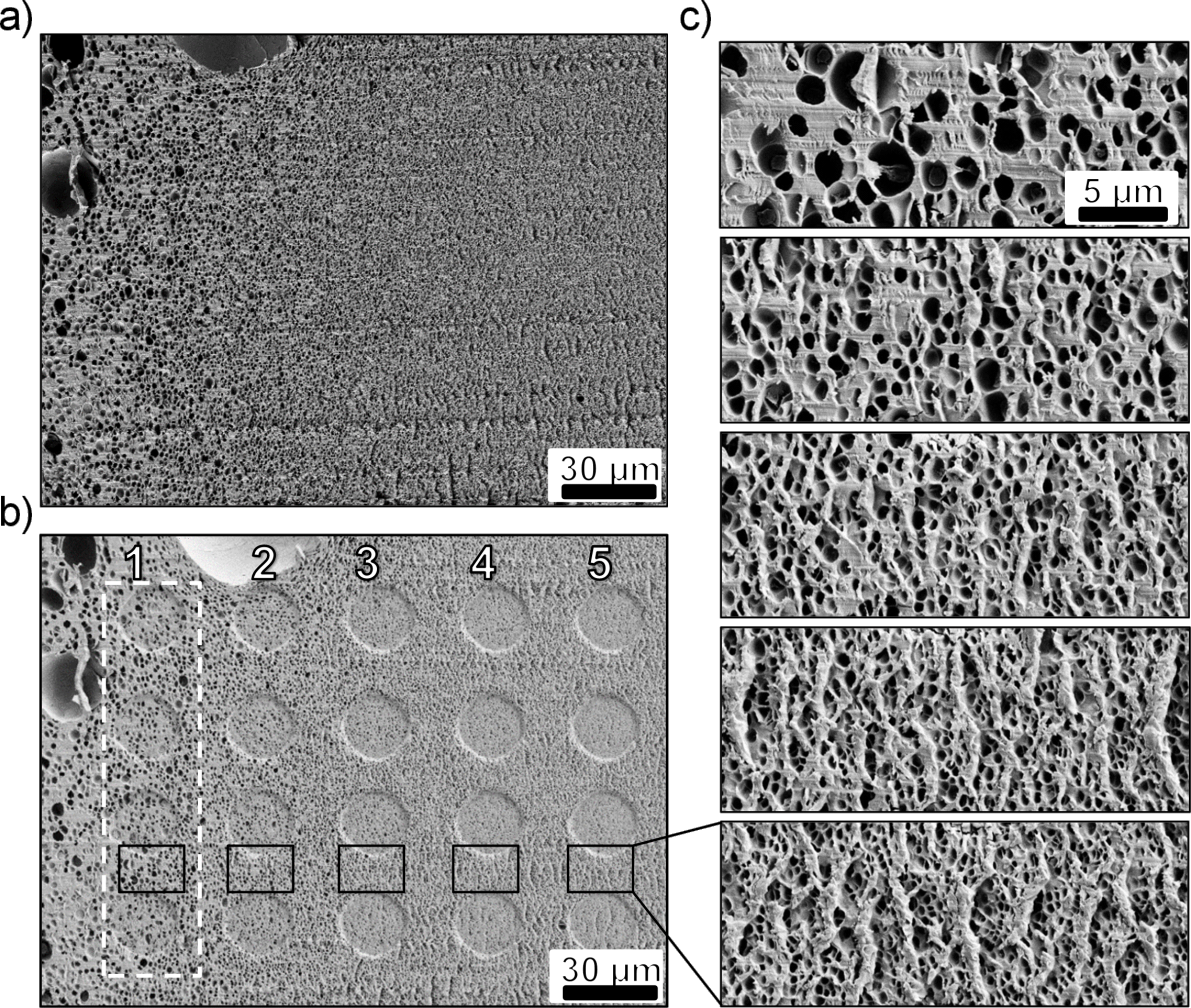
SEM images of the cross section with a gradient of pores before and after indentation. (a) The original surface before indentation. (b) The same sample position after indentation. The complex modulus was measured at 20 locations organised in a 5 × 4 array. The residual imprints of the flat punch are clearly visible. We numbered the columns from 1 (larger pore size and wall thickness) to 5 (smaller pores and thinner walls). (c) SEM images of the areas taken for the morphological analysis. Their positions are marked as black frames in panel b.

The samples were characterised by using scanning electron microscopy (SEM, SUPRA 60 VP, Zeiss). Prior to SEM analysis the samples were sputtered with silver for 100 s at 25 mA with a sputter-coater (K575X, Emitech) in order to avoid charging effects and ensure a good resolution. SEM images of the surface of interest were taken before and after the indentation experiments. For the quantitative analysis of the porous surfaces, only the undeformed regions between the indents were used (see below in Figure 2). The open-source software ImageJ [23] was used for automated analysis of pore-size and pore-area fraction. Thickness of polymeric walls was determined with the help of the recently created ND ImageJ plugin [24]. During the analysis, each pore was fitted with a sphere with the centre coordinates (*X*, *Y*) and radius *R*. The mean thickness of the polymer wall between the pore of interest *I* and the *N* closest pores is defined as


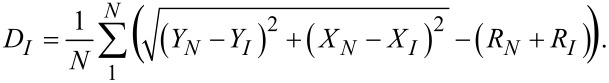


Based on the SEM images the coordination number *N* was chosen to be equal to 3.

### Preparation of cross sections

The porous PMMA films were fractured perpendicular to the surface in the middle of the sample. In order to obtain a surface of sufficient quality for nanoindentation testing, the samples were cooled with liquid nitrogen for 180 s, then broken with tweezers, fixed within a holder for nanoindentation and finally trimmed with a microtome (Leica Ultracut UCT). The areas suitable for nanoindentation experiments were determined from SEM images (see below in Figure 2a).

### Nanoindentation

For the current study, two porous PMMA samples exhibiting a structural transition from microcellular to nanocellular were prepared and their viscoelastic properties were determined in the glassy regime by dynamic nanoindentation (Nanoindenter G200 XP, Agilent/Keysight Technologies Inc., USA) or that we utilized an approach of Hay and Herbert [25] as described by Weyand et al. [26]. Briefly, a flat diamond punch of 20 μm diameter was brought into contact with the material and loaded to reach a penetration depth of 3 μm. After a stabilisation period, the punch then oscillated at different frequencies between 1 and 45 Hz while maintaining the depth of 3 μm. The frequency-specific characterisation routine yields the complex modulus E^*^ = *E'* + i*E''* as a function of the frequency for a specific indentation, with *E'* and *E''* being the storage modulus and the loss modulus of the material, respectively. The ratio *E''*/*E'*, also known as the loss factor tan δ = α, represents the overall damping capability of the material. All tests were conducted at room temperature (25.5 °C) under ambient conditions. The measurements were performed at 20 different positions arranged in an array of 5 by 4 (see below in Figure 2b).

## Results and Discussion

Porous PMMA films were produced via a modified pressure-induced saturation [20] with supercritical CO_2_, including the following steps: (i) saturating the PMMA with CO_2_, (ii) rapid depressurizing, (iii) pore growth, stimulated by heating, and (iv) fixing the structure by cooling. The nuclei of the future pores appear during the step (ii) as a result of supersaturation, as the solubility of CO_2_ in PMMA drops together with the pressure. The further growth of nuclei during the step (iii) is caused by diffusion of CO_2_ from the polymer matrix. The gradient in pore size was achieved by combining two ideas, namely to limit the penetration of CO_2_ into the sample and to employ a temperature gradient during the pore growth. The foaming process is schematically shown in Figure 1.

The glass-transition temperature (*T*_g_) of the utilized 500 μm thick PMMA sheets is 105 °C at atmospheric pressure (measured by TGA). However, because of a great plastification effect of SC-CO_2 _*T*_g_ is greatly suppressed down to room temperature during the saturation process [20] allowing for pressure-induced foaming. The resulting microstructure of the foam depends on the local concentration of SC-CO_2_. The penetration of SC-CO_2_ into the polymer is a diffusive process and can, therefore, be controlled as a function of the time and the temperature if the pressure is kept constant. It was reported previously [20] that for PMMA samples with similar dimensions, the time to complete saturation in supercritical CO_2_ is approximately 22 h. In order to achieve gradients in the pore morphology, in our approach this time span was reduced to 4 h at a saturation pressure of 30 MPa and a temperature of 44 °C. The pores first occur during a rapid drop of pressure with the condition *T > T*_g_ and the process stops with vitrification, i.e., returning the polymer to its glassy state, at lower pressure. Directly after rapid depressurisation, the samples were shortly dipped into a hot liquid with *T* ≈ 105 °C. Therefore, no equilibrium temperature could be reached. The coordinate-dependent difference Δ*T* = *T* − *T*_g_, which determined the local formation of pores, showed a gradient over the sample, being higher on the surface and lower in the bulk. We finally stopped the pore growth by cooling the sample in cool liquid as described in the Experimental section. When the described process is successful, the color of PMMA changes from transparent to white due to multiple scattering of light at the pores. A scanning electron microscopy (SEM) image of a cross section of a PMMA porous film with a gradient of mean pore diameters from 5 μm to 200 nm is presented in Figure 1.

We studied the local viscoelastic properties of the graded pore structure by dynamic flat-punch nanoindentation. This method was successfully used to characterize the influence of the microstructure of a new type of polyurethanes on their mechanical properties [26] and is, therefore, promising to characterize the graded porous materials. SEM images of the porous surface before and after nanoindentation are shown in Figure 2. Before the indentation, the surface is flat and the only visible roughness artefacts are horizontal lines, which likely occurred during trimming with the microtome. The size of pores changes gradually from several nanometres to several micrometres. The bigger pores are located in the direction to the centre of the film, indicating that CO_2_ has reached this area in smaller concentration during the saturation stage. The indentation pattern consists of an array of five columns and four rows which are easily recognizable in Figure 2b. The pore size and density are comparable over one column. Thus, an analysis of pore morphology was done based on one SEM image per column with an area of ca. 310 μm^2^ as presented in Figure 2c and marked by black frames in Figure 2b. The studied porous surface indeed exhibits a smooth gradient in its morphological characteristics. From column 1 to 5 the number of pores per SEM image gradually increases from 110 to 624, while the pore-area fraction decreases.

**Figure 3:**
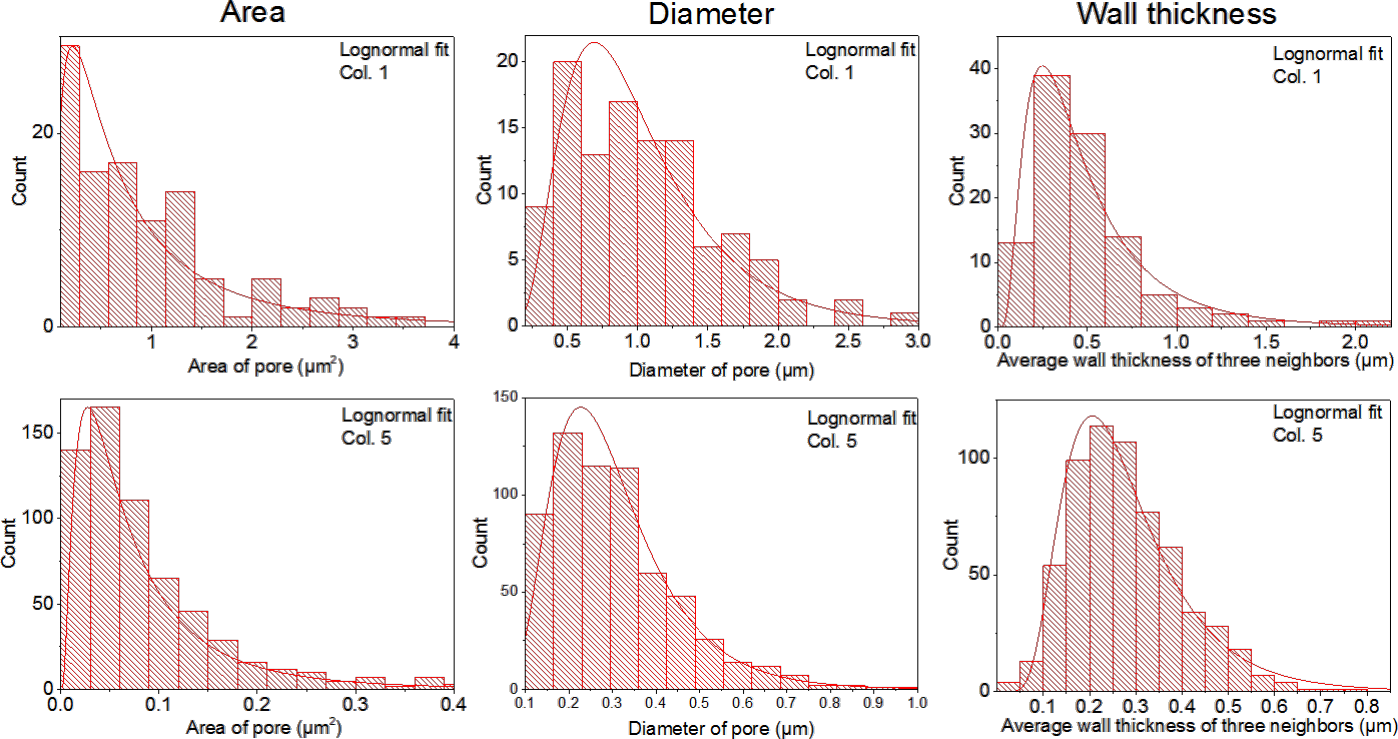
Pore distribution for the indentation columns 1 (top) and 5 (bottom). All presented data fit to a log-normal distribution and satisfy a Kolmogorov–Smirnov test for the goodness of the fits with 5% level. Column one corresponds to the biggest pores within the investigated area, the column five to the smallest ones.

The distributions of pore area, diameter and thickness of polymeric walls fit to a log-normal distribution [27] (Figure 3 and Table 1) with the exception of the wall thickness of column 4, which fits better to a gamma distribution (see Figure S1 in Supporting Information File 1). All distributions satisfy the Kolmogorov–Smirnov goodness test with a 5% level. In order to make the wall thickness of column 4 comparable with the other data sets, the characteristic values of a fitted log-normal distribution are added to Table 1 as well. By definition, a log-normal distribution cannot be negative or have a zero value, which is inevitable for the application to porous polymers. In micro- and nanocellular foams there is a critical pore radius, i.e., a minimum pore size that can exist in the fixed foam. In order to account for this minimum pore size, Ramesh et al. [28] introduced a cut-off to a log-normal distribution of pore sizes, so the resulting distribution is cut around zero-values. As the pores were analysed based on automated image processing, we introduced a minimum detectable pore size of 0.01 μm^2^, which served as the cut-off for the log-normal distribution.

**Table 1:** Properties of the porous PMMA. Pore area, diameter and wall thickness for the indentation columns 1–5, fitted both with log-normal and normal statistical models, and corresponding size-dependent values of storage modulus *E'*, loss modulus *E''* and size-independent loss factor α.

	column 1			column 2			column 3			column 4			column 5		

pores per SEM image (approx. 310 μm^2^)	110			216			397			540			624		
pore fraction, %	35.4			25.28			18.66			17.94			17.31		

log-normal distribution															
	μ	μ − σ	μ + σ	μ	μ − σ	μ + σ	μ	μ − σ	μ + σ	μ	μ − σ	μ + σ	μ	μ − σ	μ + σ

pore area, μm^2^	0.64	0.227	1.803	0.281	0.126	0.628	0.097	0.035	0.271	0.068	0.025	0.182	0.06	0.025	0.146
pore diameter, nm	902	365	613	598	198	296	351	141	236	294	115	188	277	99	155
wall thickness, nm	399	199	397	367	141	228	333	122	193	273	100	158	254	94	149

normal distribution															

pore area, μm^2^	1.041	−0.045	2.127	0.377	0.08	0.674	0.151	0.012	0.29	0.107	−0.004	0.218	0.089	−0.002	0.18
pore diameter, nm	1513	990	2036	693	449	937	439	232	646	369	189	549	337	212	462
wall thickness, nm	490	150	830	410	220	600	360	220	500	300	180	420	280	160	131

indentation results (at 45 Hz)															

storage modulus *E'*, MPa	1407 ± 30			1334 ± 29			1261 ± 23			1202 ± 12			1190 ± 19		
loss modulus *E''*, MPa	101 ± 8			102 ± 5			92 ± 10			85 ± 4			85 ± 6		
loss factor α	0.0715 ± 0.005			0.0763 ± 0.003			0.0733 ± 0.005			0.0725 ± 0.004			0.0718 ± 0.002		

**Figure 4:**
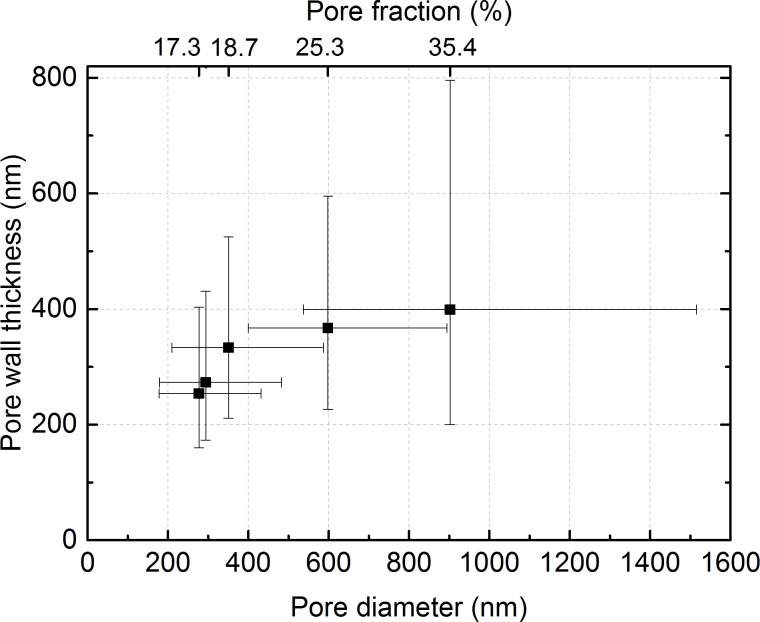
Relation between pore wall thickness, pore diameter, and pore fraction in micro-to-nanocellular PMMA. In the prepared porous sample all mentioned parameters decrease during the transition from macro- to nanocellular PMMA.

Within the analysed area, the mean pore diameter and pore area decrease by a factor of 3.3 and 10, respectively. The decreasing pore size from the microcellular to the nanocellular foam over the sample thickness results into a continuous reduction of the pore wall thickness, which is here defined as the mean distance between three neighbouring pores (see Experimental section). The nanocellular foam also features a lower pore-area fraction than the microcellular foam (Figure 4). The mean wall thickness (Table 1) decreases from 399 to 254 nm for pore area fractions of 35% and 17%, respectively. A decreasing pore-wall thickness may stimulate the confinement of the polymer chains within the pore walls and lead to a reduced mobility of the macromolecules, as it was described for PMMA-based low-density nanoporous materials with mean pore size of 200 nm [29]. However, the pores in our sample are well above this size. Therefore, we do not expect to observe such a confinement effect.

Although the cell size in cellular materials fit to a log-normal [28,30–32] or gamma [32] distribution, the arithmetical mean μ and the standard deviation σ of the pore-size distribution are also applied in various studies [21,33–35]. In order to keep our results comparable, the average dimension parameters and their standard deviations are also given in Table 1. The values of the arithmetic mean (μ) are always bigger than those of the geometric mean. However, the overall relation between the values does not change with the statistical model.

**Figure 5:**
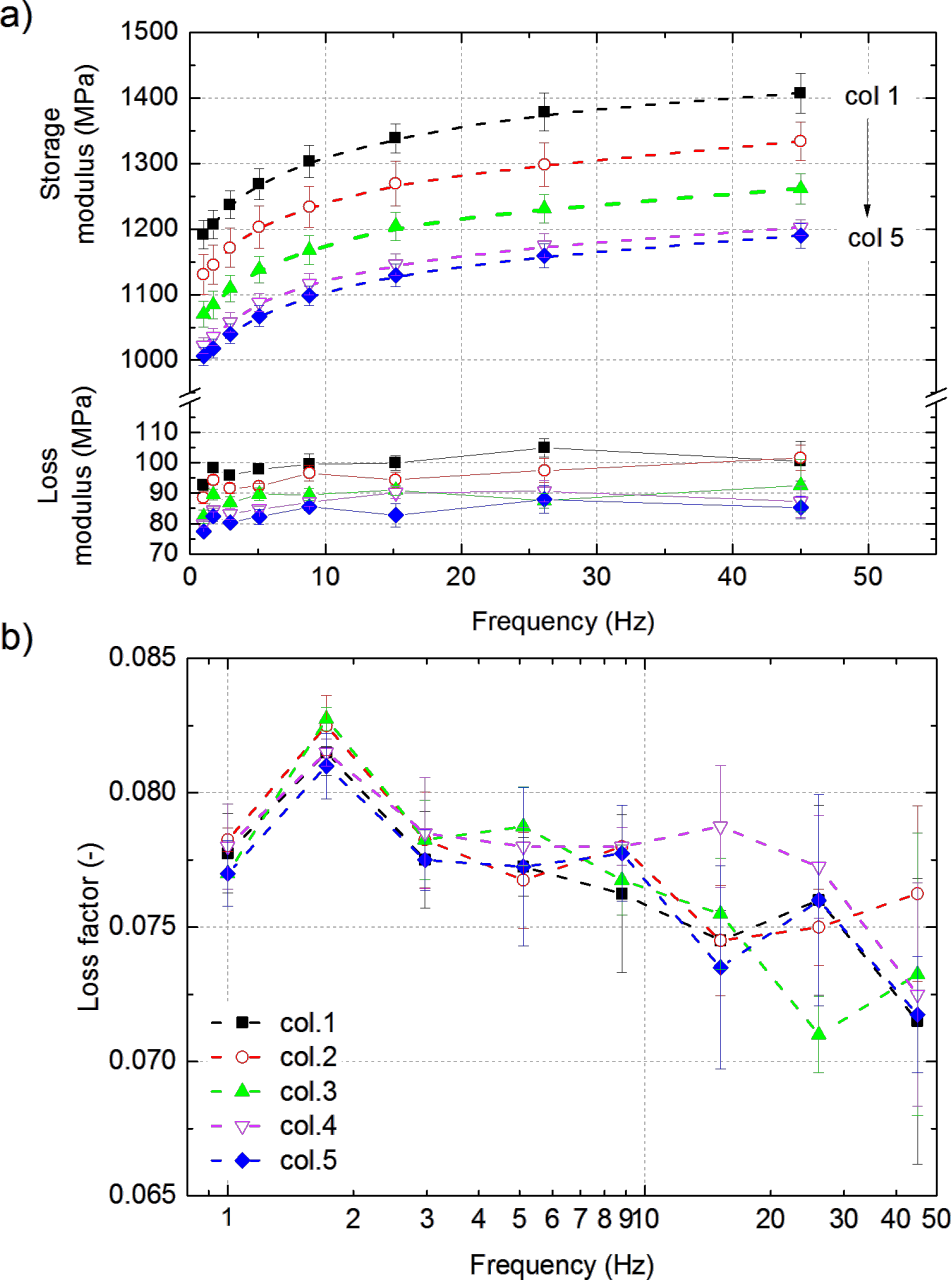
Storage and loss moduli (a) and loss factor (b) measured on the 5 × 4 arrays with flat-punch indentation. An oscillation amplitude of 50 nm and a precompression of 3 μm were applied. The measurements were taken at eight different frequencies in the range from 1 to 45 Hz. Each data point represents the mean for one column per frequency. The storage modulus decreases with column number, reaching 41% of the value for untreated PMMA. The loss factor shows no trend regarding the effect of microstructural parameters.

The values for storage modulus and loss modulus as well as the loss factor, determined from nanoindentation performed in the glassy regime, are shown in Figure 5. The storage modulus of neat PMMA measured with nanoindentation is comparable to the bulk elastic modulus provided by the manufacturer (2800 MPa and 3300 MPa, respectively) and with other published values [21,36–37]. Storage modulus and loss modulus clearly depend on the measurement location and they decrease for every measured frequency within the morphological transition from micro- to nanoporous foam. In the region with the smallest pore size (column 5), the storage modulus was 41% of the value for untreated PMMA (2800 MPa measured with the identical setup). This value is approximately 15% smaller compared to the region with the biggest pores. The loss factor is low, as expected for a stiff polymer, and in the range typical for PMMA [38]. However, there is no trend regarding the effect of microstructural parameters; within the scatter of the data, the values can be regarded to be constant over the sample thickness. The damping capability at room temperature is dominated rather by the material than the structure. Similar findings were reported for nanoporous PMMA with a relative density of 40% and pore sizes of approx. 200 nm and approx. 300 nm [21]. The mechanical properties of a foam usually depend on the relative density of the foam, which is not accessible here, since the microstructure changes gradually over the sample thickness. We observe the following dependencies: The storage modulus increases with increasing pore-area fraction, which appears counterintuitive at first sight. But for our samples, areas with larger pore area fraction also exhibit more material between the cells (see Table 1), which is expected to result in an increased modulus [38]. Following the same line of argument, the loss modulus can also be expected to decrease within the morphological transition from micro- to nanoporous foam, as can be seen in Figure 5a. This results in a loss factor that does not depend on the pore size, but rather reflects the damping capability of PMMA (Figure 5b).

**Figure 6:**
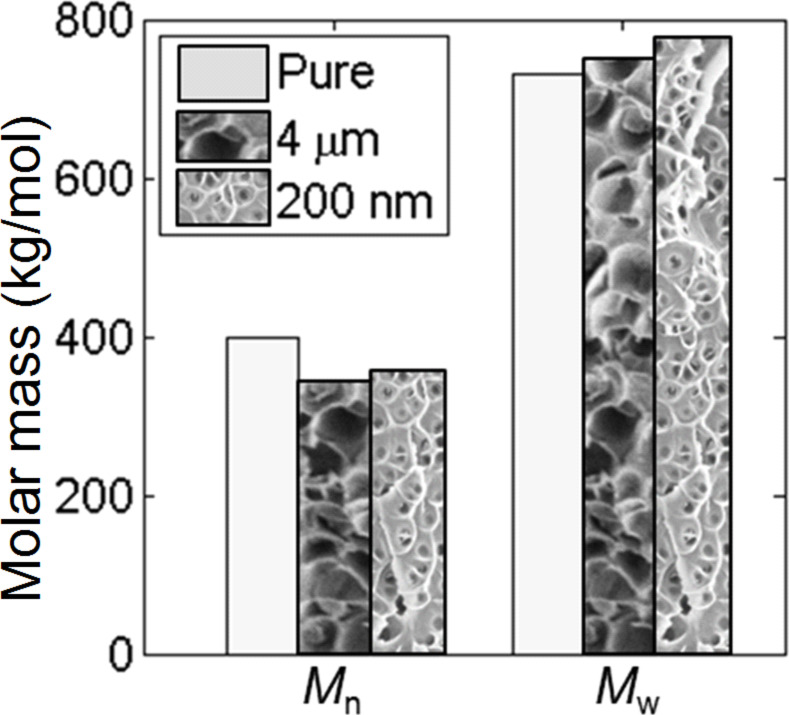
Change of the number- (*M*_n_) and mass- (*M*_w_) average of the molar mass in PMMA before and after foaming measured by size exclusion chromatography. The mean pore size is indicated in the legend. Decreasing *M*_n_ values and increasing *M*_w_ values suggest a shortening of PMMA macromolecules with lower molecular mass.

The decreasing storage modulus might also be caused by a partial damage of PMMA material and the shortening of the polymeric chains. In order to test this hypothesis, the average molar mass (*M*_n_) and the mass-average molar mass (*M*_w_) of two pieces of PMMA foam with a similar pore area fraction of 40% and different pore sizes of 4 μm and 200 nm were determined via size exclusion chromatography. An untreated PMMA sample was used as a reference. The pore area fraction and pore size were determined by SEM. The porous samples, independently on the pore diameter, show a trend of decreasing *M_n_* (by 9–10%, Figure 6). This suggests the shortening of a part of macromolecules. At the same time *M*_w_ slightly increases from 2 to 4%, suggesting that only shorter polymeric chains shortened further, while the longer ones remained intact.

The obtained data allows us to estimate the size of the average macromolecule in PMMA. Based on the *M*_w_ value of 750.000 g/mol and a weight of a single molecule of 100 g/mol, each PMMA chain contains on average 7500 MMA monomer elements. During the process of saturation with SC-CO_2_, the PMMA chains undergo a transformation from a coil shape to a globule shape and back to the coil shape after foaming [39]. Assuming that the PMMA chains have the shape of a statistical coil, we calculated from volume considerations that the coil radius should be bigger than 290 nm [40]. This value is comparable to the mean wall thickness, which, as estimated from the SEM images (Figure 6c), changes from 399 to 254 nm for areas with the biggest and smallest pores, respectively. For critically thin polymeric walls a great reduction of the elastic modulus in comparison to its bulk value was predicted from the point of discontinuous molecular dynamics [22]. We have estimated this critical value for PMMA as 30 nm, taking into account a Kuhn length of 1.53 nm [41]. Thus, in our study, the mean wall thickness is 8–13 times greater than the critical value.

## Conclusion

We analysed size-dependent viscoelastic properties of polymers at the nanoscale in the glassy regime. Local hardening and softening of the polymer at the nanoscale were predicted, but so far they were not confirmed experimentally. At the macro- and micro-scale, graded materials exhibit improved impact resistance. However the size-dependent mechanical properties of the graded materials with morphological gradients at the micro- and nano-scale have not been yet investigated. Therefore, we manufactured porous PMMA films, exhibiting a gradual change of pore size from the micro- to the nanometre range. Such a film with changing pore size was obtained by saturation in supercritical carbon dioxide under conditions of inhomogeneous pressure and temperature.

We characterised the obtained morphological gradients in terms of pore size and area fraction and performed dynamic nanoindentation on those areas. To the best of our knowledge, this is the first experimental demonstration of size-dependent viscoelastic properties of porous polymers with morphological gradient. Despite a two times lower pore-area fraction, the nanocellular region of the sample exhibited a 15% larger reduction of the storage modulus in comparison with the microcellular region. This seems contrary to the common-sense expectations at first sight, but can be rationalised by the smooth reduction of the thickness of the polymer walls. Such a gradual change of the viscoelastic properties as observed here for PMMA foams demonstrates a promising method to tailor elastic properties and achieve a stiffness gradient in other thermoplastic polymers without chemical modifications. Such stiffness gradients in thermoplastic polymers can be used in various applications, for example as passive layers for impact-resistant components [2], as an active layer for local softening of the surface of dry adhesives [5] or as a separation membrane for various chemical syntheses [42].

## Supporting Information

File 1 Additional experimental data.

## References

[1] Fratzl P, Weinkamer R (2007). Prog Mater Sci.

[2] Thielen M, Schmitt C N Z, Eckert S, Speck T, Seidel R (2013). Bioinspiration Biomimetics.

[3] Studart A R, Erb R M, Libanori R, Kim C-S, Randow C, Sano T (2015). Bioinspired Hierarchical Composites. Hybrid and Hierarchical Composite Materials.

[4] Thielen M, Speck T, Seidel R (2013). J Mater Sci.

[5] Sahay R, Low H Y, Baji A, Foong S, Wood K L (2015). RSC Adv.

[6] Maca K, Dobsak P, Boccaccini A R (2001). Ceram Int.

[7] Put S, Vleugels J, Van der Biest O (2003). Acta Mater.

[8] Colombo P, Hellmann J R (2002). Mater Res Innovations.

[9] Goetz J, Tan H, Tovar A, Renaud J (2011). SAE Int J Mater Manuf.

[10] Corbin S F, Zhao-jie X, Henein H, Apte P S (1999). Mater Sci Eng, A.

[11] Yao J, Barzegari M R, Rodrigue D (2010). Cell Polym.

[12] Moon J, Caballero A C, Hozer L, Chiang Y-M, Cima M J (2001). Mater Sci Eng, A.

[13] Fischer S F, Thielen M, Weiß P, Seidel R, Speck T, Bührig-Polaczek A, Bünck M (2014). J Mater Sci.

[14] Tomasko D L, Li H, Liu D, Han X, Wingert M J, Lee L J, Koelling K W (2003). Ind Eng Chem Res.

[15] Safford M M, Sargent D E (1968). Producing microporous polymers.

[16] Colton J S, Suh N P (1987). Polym Eng Sci.

[17] Notario B, Pinto J, Rodriguez-Perez M A (2016). Prog Mater Sci.

[18] Cooper A I (2000). J Mater Chem.

[19] Shieh Y-T, Su J-H, Manivannan G, Lee P H, Sawan S P, Dale Spall W (1996). J Appl Polym Sci.

[20] Goel S K, Beckman E J (1994). Polym Eng Sci.

[21] Notario B, Pinto J, Rodríguez-Pérez M A (2015). Polymer.

[22] Böhme T R, de Pablo J J (2002). J Chem Phys.

[23] Schneider C A, Rasband W S, Eliceiri K W (2012). Nat Methods.

[24] Haeri M, Haeri M (2015). J Open Res Software.

[25] Hay J, Herbert E (2013). Exp Tech.

[26] Weyand S, Blattmann H, Schimpf V, Mülhaupt R, Schwaiger R (2016). Mater Res Express.

[27] Jolicoeur P (1999). The lognormal distribution. Introduction to Biometry.

[28] Ramesh N S, Rasmussen D H, Campbell G A (1994). Polym Eng Sci.

[29] Reglero Ruiz J A, Dumon M, Pinto J, Rodriguez-Pérez M A (2011). Macromol Mater Eng.

[30] Feng J J, Bertelo C A (2004). J Rheol.

[31] Richardson J T, Peng Y, Remue D (2000). Appl Catal, A.

[32] Vecchio I, Redenbach C, Schladitz K, Kraynik A M (2016). Comput Mater Sci.

[33] Laguna-Gutierrez E, Van Hooghten R, Moldenaers P, Rodriguez-Perez M A (2015). J Appl Polym Sci.

[34] Pinto J, Dumon M, Pedros M, Reglero J, Rodriguez-Perez M A (2014). Chem Eng J.

[35] Limpert E, Stahel W A, Abbt M (2001). BioScience.

[36] Léonardi F, Allal A, Marin G (2002). J Rheol.

[37] O'Rourke Muisener P O, Clayton L, D’Angelo J, Harmon J P, Sikder A K, Kumar A, Cassell A M, Meyyappan M (2002). J Mater Res.

[38] Ashby M F, Cebon D (1993). J Phys IV.

[39] Meredith J C, Johnston K P (1998). Macromolecules.

[40] Bhushan B (2010). Springer Handbook of Nanotechnology.

[41] Kuhlman W A, Olivetti E A, Griffith L G, Mayes A M (2006). Macromolecules.

[42] Mulder J (1996). Basic principles of membrane technology.

