# Supplementary material for: Bio-inspired micro-to-nanoporous polymers with tunable stiffness
Source: Beilstein J Nanotechnol. 2017 Apr 21;8:906–14. doi: 10.3762/bjnano.8.92 (PMC5405679; doi:10.3762/bjnano.8.92)
Supplement: File 1 — Additional experimental data. [file Beilstein_J_Nanotechnol-08-906-s001.pdf]

Supporting Information

for

Bio-inspired micro-to-nanoporous polymers with tunable stiffness

Julia Syurik[1], Ruth Schwaiger[2], Prerna Sudera[1], Stephan Weyand[2], Siegbert Johnsen[3], Gabriele Wiegand[3] and Hendrik Hölscher*[1]

Address: [1]Institute for Microstructure Technology, Karlsruhe Institute of Technology (KIT), Hermann-von-Helmholtz-Platz 1, 76344 Eggenstein-Leopoldshafen, Germany; [2]Institute for Applied Materials, Karlsruhe Institute of Technology (KIT), Hermann-von-Helmholtz-Platz 1, 76344 Eggenstein-Leopoldshafen, Germany and [3]Institute of Catalysis Research and Technology, Karlsruhe Institute of Technology (KIT), Hermann-von-Helmholtz-Platz 1, 76344 Eggenstein-Leopoldshafen, Germany

Email: Hendrik Hölscher* - hendrik.hoelscher@kit.edu

* Corresponding author

Additional experimental data

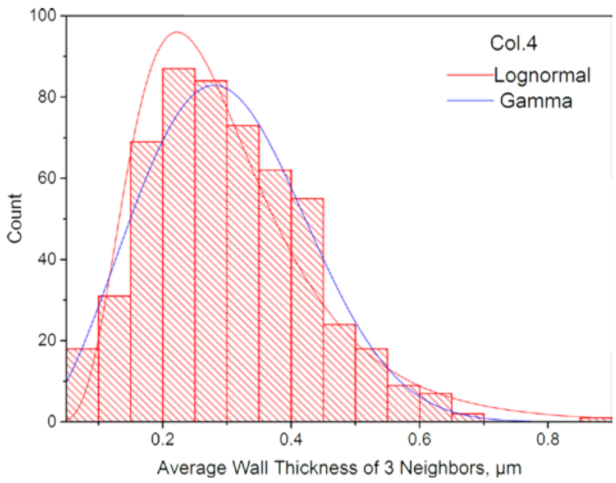

Figure 1: Distribution of the average distance between pores for the indentation column four. The data is fitted with a log-normal distribution (red solid line), which does not satisfy a Kolmogorov–Smirnov test at 5% level with a P-value of 0.03, and with Gamma distribution (blue solid line), which satisfies the Kolmogorov–Smirnov test at 5% level with a P-value of 0.20. In order to have comparable values for all indentation columns, the log-normal distribution was used in the study.
